# Xinyang tablet ameliorates sepsis-induced myocardial dysfunction by regulating Beclin-1 to mediate macrophage autophagy and M2 polarization through LncSICRNT1 targeting E3 ubiquitin ligase TRAF6

**DOI:** 10.1186/s13020-023-00832-7

**Published:** 2023-11-02

**Authors:** Yuanyuan Luo, Yuanmei Li, Liwei He, Haitao Tu, Xinfeng Lin, Fengli Zhao, Yusheng Huang, Minyong Wen, Lingjun Wang, Zhongqi Yang

**Affiliations:** 1 Department of Intensive Care Unit, The First Affiliated Hospital of Guangzhou University of Chinese Medicine, Guangzhou, China; 2 Department of Rehabilitation Medicine, Nanfang Hospital of Southern Medical University, Guangzhou, China; 3 Department of Cardiology, Nanfang Hospital of Southern Medical University, Guangzhou, China; 4 Department of Nephrology, The First Affiliated Hospital of Guangzhou University of Chinese Medicine, Guangzhou, China; 5 Lingnan Medical Research Center, Guangzhou University of Chinese Medicine, Guangzhou, China; 6 President‘s Office, The First Affiliated Hospital of Guangzhou University of Chinese Medicine, Guangzhou, China

**Keywords:** Xinyang tablet, Sepsis-induced myocardial dysfunction, LncSICRNT1, TRAF6, Macrophages, M1/M2 polarization, Beclin-1, Ubiquitination

## Abstract

**Objective:**

Xinyang Tablet (XYT) has emerged as a potential intervention to counter sepsis-induced myocardial dysfunction (SMID) by influencing macrophage autophagy and M2 polarization. This study aimed to unravel the underlying mechanism of XYT in sepsis-induced myocardial dysfunction (SIMD).

**Methods:**

A microarray analysis was employed to explore sepsis-related changes, and bioinformatics analysis was used to predict lncRNAs binding to tumor necrosis factor receptor-associated factor 6 (TRAF6). This studio utilized SIMD mouse models induced by lipopolysaccharide (LPS) injection, followed by treatments involving varied doses of XYT, digoxin (positive control), or si-LncSICRNT1. After seven days, evaluations encompassing mouse hair/mental state/diet/weight were measured, and cardiac function via echocardiography were conducted. Myocardial tissue changes were observed using hematoxylin–eosin staining. Additionally, bone marrow-derived macrophages (BMDMs) subjected to LPS for M1 polarization were treated with oe-LncSICRNT1, si-TRAF6 and their negative control, XYT, or autophagy inhibitor 3-Methyladenine (3-MA) (positive control). RT-qPCR and Western blot analyses were employed to assess LncSICRNT1, TRAF6, Beclin-1, LC3II/LC3I, and p62 levels. Immunohistochemistry and flow cytometry were used for M1/M2 polarization markers, while enzyme-linked immunosorbent assay (ELISA) gauged inflammatory factor levels. Interaction between TRAF6 and LncSICRNT1 was probed using RNA pull-down and RNA immunoprecipitation (RIP) assays.

**Results:**

Chip analysis obtained 1463 differentially expressed lncRNAs, including LINC01550 (LncSICRNT1). Further prediction indicated that LncSICRNT1 was highly likely to directly bind to TRAF6. XYT treatment in LPS-induced SIMD mice led to notable enhancements in sleep/hair/diet/activity, increased weight/left ventricular end-diastolic diameter (LVEDd)/LV ejection fraction (LVEF)/LV fraction shortening (LVFS). These improvements were associated with elevated LncSICRNT1 expression and decreased TRAF6 protein levels, culminating in reduced myocardial inflammatory responses and improved cardiac function. Notably, XYT was found to suppress macrophage M1 polarization, while enhancing M2 polarization, ultimately benefitting cardiac function via LncSICRNT1 modulation. Furthermore, the study revealed LncSICRNT1 modulated Beclin-1 ubiquitination and restrained macrophage autophagy by targeting TRAF6 expression.

**Conclusion:**

The study highlights XYT’s potential to ameliorate LPS-induced SIMD by elevating LncSICRNT1 expression, influencing TRAF6 expression, and regulating Beclin-1 ubiquitination. These actions collectively inhibit macrophage autophagy and foster M1/M2 polarization, contributing to cardiac function improvement.

**Supplementary Information:**

The online version contains supplementary material available at 10.1186/s13020-023-00832-7.

## Introduction

Sepsis, a grave condition, arises when the body reacts poorly to infection, causing organ dysfunction [1]. Annually over 30 million people worldwide are diagnosed with sepsis, leading to about 5 million deaths [2]. Sepsis results in systemic hypotension, inflammation, and compromised organ function [3]. The heart is often the initial organ affected by sepsis, impacting over 40% of patients and contributing to a mortality rate of up to 70% [4], [5]. Swift diagnosis and treatment are imperative. Sepsis-induced myocardial dysfunction (SIMD), a reversible cardiac issue caused by sepsis, is characterized by ventricular enlargement, reduced compliance, and diminished responsiveness [6]. Despite established sepsis treatment guidelines [7, 8], currently there is no specific treatment for SIMD due to its multifaceted etiology.

The innate immune system is central to the defense responses in sepsis, aiming to eliminate invasive microorganisms and triggering potent acute inflammatory reactions [9]. It is noteworthy that macrophages are pivotal essential components of innate immunity, playing a dominant role in inflammation and host defense [10, 11]. Macrophages demonstrate diversity and adaptability, with M1 macrophages activated by Toll-like receptor (TLR) ligands and interferon (IFN)-γ, while M2 macrophages respond to interleukin (IL)-4 and/or IL-13 [12]. Macrophage serve as the primary source of inflammatory cytokines, which is crucial during sepsis-induced cardiomyopathy [13]. M1-polarized macrophages largely drive sepsis, underscoring the significance of restraining M1 polarization and fostering M2 polarization for effective sepsis management [14]. The aforementioned evidence underscores the considerable importance of macrophage polarization in SIMD.

In recent years, traditional Chinese medicine has gained substantial attention as a treatment for heart failure and has been recognised as a significant advancement by the *Journal of the American College of Cardiology* [15]. One such treatment is Xinyang Tablet (XYT), an authorized formulation by the Guangdong Pharmaceutical and Food Administration (No. Z20071257), containing a blend of ingredients (including *Radix astragali*, *Herba epimedii*, *Semen lepidopteris*, red ginseng, motherwort, holly, and Chinese plantain), which has been clinically applied for nearly 20 years has demonstrated its efficacy against heart failure by inhibiting myocardial apoptosis and inflammation under pressure load [16]. Notably, the active compound astragalus polysaccharide from astragalus membranaceus, a component of XYT, is recognised for its immunomodulatory effects [17]. This compound facilitates macrophage M2 polarization and improves vascular endothelial dysfunction by boosting the nuclear factor erythroid 2-related factor 2/Heme oxygenase-1 pathway [18]. Another crucial bioactive compound, astragaloside IV, found in astragalus membranaceus, modulates macrophage phenotype via the STAT signaling reconfiguration, providing partial relief in experimental DSS-induced colitis [19]. Besides, the active ingredients icariin and PEG hydrogel in Epimedium induce macrophage polarization to M2 type [20]. In LPS-stimulated mouse macrophages, anhydrous icariin pretreatment markedly lowers iNOS protein expression ^21^. Stachydrine and Leonurine, the primary active components of motherwort, have been reported to possess anti-inflammatory and antioxidant functions [22, 23]. Leonurine can down-regulate IL-1β and TNF-α, and impede the NLRP3 inflammasome activation and macrophage M1 polarization [24]. Based on these findings, we hypothesize that XYT mild hold potential in alleviating SIMD.

Long non-coding RNAs (lncRNAs), consisting of more than 200 nucleotides, play significant roles at epigenetic, transcriptional, and post-transcriptional levels, contributing to various biological process such as inflammatory responses, age-associated cardiovascular aging, and cancer biology [25]. Notable, lncRNAs facilitate the shift of macrophages towards the M2 phenotype through modulation of specific signaling pathways. They act as transcription factors decoys and miRNA sponges, crucially mediating macrophage polarization in response to extracellular or intracellular stimuli [26]. Moreover, there is evidence suggesting that astragalus membranaceus can influence the expression of the lncRNA-mRNA network [27]. Moreover, there has been evidence that Astragalus membranaceus is capable of modulating the expression of the lncRNA-mRNA network [27]. The expression of lncRNA H19 and lncRNA CYTOR can be modulated by icariin [28, 29]. Hence, it is reasonable to speculate that XYT may enhance macrophage M2 polarization and mitigate SIMD by modulating specific lncRNAs.

A noteworthy observation is that dampening the activity of tumor necrosis factor (TNF) receptor-associated factor 6 (TRAF6) can alleviate myocardial dysfunction in LPS-induced sepsis [30]. TRAF6, functioning as an E3 ubiquitin ligase, acts as a signal transducer for inflammatory signaling [31]. More significantly, TRAF6 is involved in regulating autophagy induced by Toll-like receptor 4 (TLR4) achieved through ubiquitination of Beclin-1 [32]. Beclin-1, the mammalian homolog of yeast Vps30/Atg6, serves as a pivotal facilitator of autophagy [33]. Beclin-1 undergoes diverse post-translational modifications, including phosphorylation, ubiquitination, ISGylation, and acetylation, ultimately influencing the autophagy process [34]. Notably, autophagy is a participant in modulating macrophage polarization [35] and excessive autophagy can potentially exacerbate inflammatory responses [36]. TRAF6 mediates Lysine 63 (K63)-linked ubiquitination of Beclin-1, disrupting the Beclin1-Bcl2 interaction, promoting the interaction between class III phosphatidylinositol 3-kinase (PI3KC3) with Beclin-1, and thereby leading to an accumulation of autophagosomes in macrophages [14, 37].

Building upon the aforementioned research, we conducted bioinformatics analysis utilising the Gene Expression Omnibus (GEO) database and lncRNA prediction website. Our investigation led us to speculate that XYT could potentially enhance SIMD via regulating macrophage M2 polarization, autophagy, and Beclin-1 ubiquitination through lncRNA-LINC01550 (we named LncSICRNT1 in this text) and TRAF6. The purpose of this study was to investigate the molecular mechanism of XYT in improving SIMD through LncSICRNT1, offering substantial evidence to support the therapeutic effect of XYT on SIMD and encourage the integration of traditional Chinese medicine into clinical applications.

## Materials and methods

### Ethics statement

All experiments procedures were approved by the Animal Care and Use Committee of The First Affiliated Hospital of Guangzhou University of Chinese Medicine. The care and treatment of mice during the experimental process adhered to the guidelines outlined in the *Instructive Notions with Respect to Caring for Laboratory Animals* issued by the Ministry of Science and Technology of China in 2006.

### Bioinformatics analysis

We obtained the LncRNA microarray GSE145227 related to sepsis from the GEO database (https://www.ncbi.nlm.nih.gov/geo/). This dataset included 12 normal samples and 12 sepsis samples. Employing the Bioconductor limma package, we conducted differential analysis of lncRNA and corrected *p*-value for false discovery rate, using normal samples as controls. Differentially expressed lncRNAs were identified based on the criteria of |logFC|> 1 and adj.*p*.val < 0.05. For analysis of TRAF6, we utilized the catRAPID database (http://service.tartaglialab.com/page/catrapid_group) to analyze the amino acid sequence of TRAF6 and pinpoint the RNA-binding domain. Meanwhile, the potential binding lncRNAs of TRAF6 were predicted.

### SIMD mouse modeling and grouping

We obtained specific-pathogen-free C57BL/6 J male mice (8-week-old, weighing 19 g-23 g) from the Institute of Laboratory Animal Sciences, CAMS&PUMC (Beijing, China). The mice were acclimated under regular conditions for a week, at a temperature of 22–25 °C, a humidity of 70%, a 12/12-h light/dark cycles, and ad libitum access to food and water.

Establishment of SIMD mouse models: the mice’s abdominal skin of mice underwent disinfection with medical iodophor and the iodophor was subsequently wiped off with cotton. A single intraperitoneal injection of 10 mg/kg LPS was then administered [38]. Following the procedure, mice were allowed ad libitum access to food and water. Six hours after administration, echocardiography was performed to assess the success of the model, with LVFS < 50% as a marker of successful SIMD mouse modeling [13, 39].

Grouping: The mice were grouped randomly as follows (N = 12): (1) control group: injected intraperitoneally with 10 mg/kg normal saline at one time, with other treatments the same as the LPS group; (2) LPS group; (3) LPS + oe-LncSICRNT1 group: immediately injected with oe-LncSICRNT1 adeno-associated virus plasmids (19 × 10^7^ TU/per mouse) via tail veins after modeling [40]; (4) LPS + oe-negative control (NC) group: immediately injected with an equivalent amount of oe-NC plasmids via tail veins after modeling; (5) LPS + XYT-L, M, and H group (the mice were intragastrically administered with low-, medium-, and high-dose XYT on day 2 after modeling); (6) LPS + digoxin group (positive drug control group); (7) LPS + XYT-M + si-LncSICRNT1 group: immediately injected with si-LncSICRNT1 plasmids (19 × 10^7^ TU/per mouse) via tail veins and treated with medium-dose XYT suspension 2 days later after modeling; (8) LPS + XYT-M + si-NC group: immediately injected with equivalent amounts of si-NC plasmids and treated with medium-dose XYT suspension on the second day after modeling. All plasmids were provided by GenePharma (Shanghai, China).

The human dose of XYT, a Chinese patent medicine for invigorating Qi, warming Yang, and promoting blood circulation (prepared at The First Affiliated Hospital of Guangzhou University of Chinese Medicine), contains constituents such as red ginseng, astragalus membranaceus, epimedium, holly, motherwort, tinglizi, 0.25 g/tablet. A human intake of 4 tablets, thrice daily, equates to 43 mg/kg. By employing conversion factors outlined in the Research Methods in Pharmacology of Chinese Materia Medica, the equivalent clinical doses of XYT for mice were calculated: medium dose (387 mg/kg), low dose (194 mg/kg), and high dose (774 mg/kg). The equivalent dose of digoxin used was 32 mg/kg. The drugs were ground into powder, mixed with pure water, and formulated into a suspension for intragastric administration. Mice were given this suspension 2 times/day for 7 consecutive days, while the LPS and control groups were administered an equal volume of pure water. After the 7-day regimen observations were made regarding mouse hair, mental state, and diet. Furthermore, mouse weight was documented, and cardiac function was evaluated using echocardiography. Euthanasia was carried out using 100 mg/kg pentobarbital sodium, followed by the excision of cardiac tissues. Of these, six were embedded in paraffin for hematoxylin–eosin (HE) staining and immunofluorescence analysis, while the cardiac tissues from the remaining six mice were employed for flow cytometry, enzyme-linked immunosorbent assay (ELISA), reverse transcription-quantitative polymerase chain reaction (RT-qPCR), and Western blot. All samples were promptly frozen in liquid nitrogen and then stored at -80 °C. The in vivo experiment was shown in Additional file 1: Figure S1.

## Echocardiography

Following the administration of anaesthesia to the mice on both the 1st and 7th day post-operation, we employed high-resolution echocardiographic system (Vevo 2100, VisualSonics, Toronto, Ontario, Canada) to evaluate their cardiac function. This system was equipped with a 12 MHz linear sensor, enabling us to conduct transthoracic 2D M-mode echocardiography and pulsed Doppler spectrum analysis. The process involved placing an ultrasound probe beside the sternum of mice to capture the parasternal short-axis view of left ventricle at the level of papillary muscles. After obtaining a clear 2D image, we transitioned to M-mode echocardiography and made adjustments using the trackball. Key measurements, such as LV ejection fraction (LVEF), LVEDd, and LVESd were measured and the LVFS were derived from these evaluations. All data were collected over a span of 10 cardiac cycles by an observer who was blind to the experimental conditions.

### HE staining

HE staining was conducted using the HE staining kit (G1120-100, Solarbio, Beijing, China). The cardiac tissues of mice were cleansed with normal saline to eliminate any residual blood. Next, the apical tissues were selected and fixed with 4% paraformaldehyde for a duration of 48 h. After undergoing processes such as dehydration, clearing, and permeabilization, the tissues were embedded in paraffin, sectioned, affixed on glass slides, and toasted for 40 min. Following these steps, the prepared tissue sections were deparaffinized via xylene, dehydrated with gradient alcohol, rinsed using distilled water, and then stained with hematoxylin for about 5 min. A brief rinse under running water ensued, and sections were subjected to differentiation via 1% hydrochloric acid ethanol. After undergoing a graded alcohol dehydration process, the sections were stained with eosin. Subsequent to the completion of the dehydration and clearing steps, the sections were mounted. Utilizing an optical microscope (Olympus, Tokyo, Japan), we observed the pathological structure and morphological alterations within the myocardial tissues.

### Cell culture

The bone marrow-derived macrophages (BMDMs) were obtained as previously described [41, 42]. Briefly, following euthanasia of the mice with pentobarbital sodium (100 mg/kg), a syringe loaded with Dulbecco's modified Eagle medium (DMEM) (IB9PC-001, I-bio, Fuzhou, Jiangxi, China) was employed to flush bone marrow cells from the tibias and femurs of the mice. These bone marrow cells were subsequently incubated in DMEM with 10% fetal bovine serum (FBS) and 10 ng/mL mouse macrophage colony-stimulating factor (Sino Biological Inc., Beijing, China) in a cell incubator with 5% CO_2_ at 37 °C for 7 days to isolate BMDMs. Next, the BMDMs were stimulated with LPS (50 ng/mL, Sigma-Aldrich, Merck KGaA, Darmstadt, Germany) for 24 h to induce the M1 macrophage phenotype [43]. As controls, BMDMs treated with an equivalent amount of phosphate-buffered saline (PBS) for 24 h were utilized.

### Cell transfection and grouping

BMDMs were cultured in double-antibody-free DMEM medium (KGM12500N-500, KeyGEN Biotech, Nanjing, Jiangsu) within 24-well plates. Upon achieving 70–80% cell confluence, the cells were transfected with oe-SICRNT1, si-TRAF6 (following transfection with si1-TRAF6 and si2-TRAF6, respectively, the more effective si1-TRAF6 was chosen for subsequent experiments), and their respective NC plasmids using Lipofectamine 2000 kits (GenePharma). Following transfection, the cell plates were incubated for 48 h at 37 °C in a 5% CO_2_ incubator. Subsequently, the cultured macrophages were transferred to 6-well plates. After a 4 h adherence period, the BMDMs were stimulated with 50 ng/mL LPS for 24 h to induce polarization towards the M1 macrophage phenotype [43]. All plasmids were procured from GenePharma.

The cells were allocated into the following groups: blank group; M1 group; M1 + oe-NC group; M1 + oe-LncSICRNT1 group; M1 + oe-LncSICRNT1 + oe-NC group; M1 + si-TRAF6 group; M1 + si-NC group; M1 + XYT group: concomitantly treated with LPS and XYT (30 μg/mL) for 24 h [16]; M1 + 3-MA group: treated with LPS and 10 mM 3-methyladenine (3-MA) [44]; and M1 + DMSO group: treated with LPS and 10 mM dimethyl sulphoxide (DMSO) for 24 h.

### RT-qPCR

Total RNA was extracted from mouse cardiac tissues or cells using TRIzol reagents (#15,596,026, Thermo Fisher Scientific, Waltham, MA, USA). Following this, 1.0 μg of total RNA was employed for microarray analysis using mouse-specific kits (#G4846A, Agilent, Santa Clara, CA, USA). To quantify gene expression, 1.0 μg of total RNA was reverse-transcribed to generate first-strand cDNA with the use of kits (#AB1453A, Thermo Fisher Scientific). RT-qPCR analysis was conducted using the SYBR Green kits (#QR0100, Sigma-Aldrich) and the primers listed in Table 1 were utilized for detecting gene expression. The relative expression levels of genes were determined based on the 2^−ΔΔCt^ method, with β-actin serving as the internal control.

### Western blot assay

Total proteins were extracted from macrophages and cardiac tissues using radio-immunoprecipitation assay (RIPA) lysis buffer (#89,900, Thermo Fisher Scientific) supplemented with a protease inhibitor cocktail (#5871, Cell Signaling Technology, Shanghai, China). The quantification of extracted proteins was conducted using bicinchoninic acid (BCA) kits (Thermo Fisher Scientific). Subsequently, the total proteins were separated through sodium dodecyl sulfate–polyacrylamide gel electrophoresis and transferred onto polyvinylidene fluoride membranes (Millipore, Billerica, MA, USA). Following a blocking step with 5% skim milk for 2 h, the membranes were incubated overnight with primary antibodies anti-TRAF6 (1:2000, ab33915, Abcam, Cambridge, MA, USA), anti-Beclin-1 (1:2000, ab207612, Abcam), anti-light chain 3 (LC3; 1:2000, ab192890, Abcam), and anti-p62 (1:10,000, ab109012, Abcam) at maintained at a temperature of 4 °C. Following the incubation, the membranes underwent 3 washes before being probed with a secondary antibody goat anti-rabbit anti-IgG (1:3000, ab6721, Abcam) for 1 h. After additional 3-washes, the membranes were exposed to a chemiluminescence solution and developed using appropriate equipment. Image J software (NIH, Bethesda, Maryland, USA) was employed for the analysis of image gray values, with GAPDH (1:2500, ab9485, Abcam) serving as an internal reference.

### RNA immunoprecipitation (RIP) assay

To investigate the interaction between RNA and protein in cells, we employed the Magna RIP kits (Millipore). When cells reached a confluence of 70–80%, they were harvested and washed with cold PBS. Subsequently, 1 × 10^7^ cells were lysed using 200 µL lysis buffer containing 0.5 µL protease inhibitor cocktail and 0.25 µL RNase inhibitor. After centrifugation to remove cell debris, protein A/G beads were incubated with anti-TRAF6 antibody (1:100, Ab137452, Abcam) or mouse IgG (1:500, ab207996, Abcam) (NC) for 30 min at 37 °C with agitation. Thereafter, the cell lysates were co-incubated with bead-protein complexes overnight at 4 °C. The TRAF6-associated RNA was subsequently separated and purified using a proteinase K buffer. The eluted co-coprecipitated RNA present in an aqueous solution underwent RT-qPCR analysis, with the corresponding primers listed in Table 1 employed to assess the enrichment of LncSICRNT1 within TRAF6 protein complexes.

**Table 1:** Primer sequences

Gene	Forward 5′–3′	Reverse 5′–3′
LncSICRNT1	AGCACGTCTCCCTTTTCTGA	ATAACAGCCACCCAAAGACG
β-Actin	CATCCGTAAAGACCTCTATGCCAAC	ATGGAGCCACCGATCCACA

### RNA pull-down assay

Utilizing the Pierce Magnetic RNA–protein Pull-Down kits (20,164, Thermo Fisher Scientific), we implemented the subsequent procedure. We biotinylated and labeled a 100 pmol RNA complementary to the LncSICRNT1 junction sequence. This biotinylated RNA was then coupled to 50 µL Streptavidin magnetic bead lysis butter. As a contrast, negative RNA control was included. Subsequently, the cell lysates were then incubated for 1 h at 4 °C with rotation, in the presence of the RNA-bound beads along with 1 × binding buffer and 50% glycerol. After undergoing three washes with ice-cold 1X washing buffer, the RNA-binding-protein were isolated and detected by Western blot.

### Immunofluorescence

To conduct the immunofluorescence assay, the cardiac tissues were sectioned into slices of 3-μm-thickness. These paraffin sections were subjected to deparaffinization using the deparaffinized agent (6,971,725,071,818, clearer), followed by hydration through gradient of ethanol concentrations of 100%, 95%, and 75%. Post deparaffinization, the sections were permeablized with 0.5% Triton X-100 for 30 min, and then blocked using 3% bovine serum albumin for 1 h. Subsequently, the sections were subjected to overnight incubation with primary antibodies anti-CD38 (1:50, ab235590, Abcam), inducible nitric oxide synthase (iNOS; 1:500, ab178945, Abcam), CD163 (1:200, ab182422, Abcam), or CD206 (1:100, ProteinTech Group, Rosemont, IL, USA), all maintained at 4 °C. Next, secondary antibody staining was conducted using goat anti-rabbit IgG H&L (Alexa Fluor® 488) (1:1000, ab150077, Abcam) for a period of 2 h. Thereafter, 4’,6-diamidino-2-phenylindole (Invitrogen) was used for counterstaining. All sections were imaged under a confocal laser scanning microscope (Olympus, Tokyo, Japan). The positively stained cells were quantified by two blinded observers using Image J software (NIH).

### Flow cytometry

Tissue homogenate was prepared from mouse myocardial tissue, which was then filtered with a 40 µm cell strainer. The resultant mixture was centrifuged at 500 × g for 5 min at 4 °C, and the supernatant was discarded. The sediment or cells were then resuspended in fluorescence-activated cell sorting buffer (00–4222, eBioscience, San Diego, CA, USA) to achieve a concentration of 2 × 10^6^/mL, thereby creating a single-cell suspension. The cells were then exposed to specific antibodies: anti-CD38 (1:500, ab235590, Abcam), anti-iNOS (1:200, ab239990, Abcam), anti-CD163 (1:60, ab182422, Abcam), or anti-CD206 (fluorescein isothiocyanate; FITC) (4 µL/10^6^ cells, ab270647, Abcam) for 30 min while kept on ice. This was followed by a 30-min incubation with secondary antibody goat anti-rabbit IgG H&L (Alexa Fluor® 488) (1:2000, ab150077, Abcam) or goat anti-mouse IgG H&L (FITC) (1:500, ab6785, Abcam). As controls, rabbit IgG (1:20, ab125938, Abcam) or mouse IgG2a (1:100, ab283345, Abcam) were included. Following 2 washes, the cells were resuspended in PBS containing 2% FBS. The macrophage phenotype was assessed using a flow cytometer (FACSAria III; BD Biosciences, San Jose, CA, USA) and the obtained data were analyzed using FlowJo (version 10.0.7r2; FlowJo LLC, Ashland, OR, USA).

### ELISA

To extract proteins, mouse cardiac tissues or macrophages were lysed with RIPA buffer containing protease inhibitors (Solarbio). Following the lysis, the samples were subjected to centrifugation at 14,000×*g* for 5 min, leading to the collection of the supernatant. The levels of inflammatory factors within tissue homogenate or cell supernatant were assessed through utilisation of ELISA kits, including proinflammatory cytokines TNF-α (MTA00B, R&D Systems, Minneapolis, MN, USA), IL-6 (ml064292, Enzyme-linked Biotechnology, Shanghai, China), and IL-1β (MLB00C, R&D Systems) and anti-inflammatory cytokines IL-10 (M1000B, R&D Systems) and IL-4 (DY404-05, R&D Systems).

### Co-immunoprecipitation (Co-IP)

To initiate cell lysis. a Nonidet P40 lysis buffer supplemented with 10 mM nicotinamide and 10 μM TSA was employed. The total protein content of the lysates was then determined using a Genesys 10 UV–Vis spectrophotometer and Pierce BCA protein assay kit (both from Thermo Fisher Scientific). Subsequently, the cell lysates was subjected to overnight incubation with antibody anti-Beclin-1 (1:2000, ab207612, Abcam) or NC IgG (1:100, ab172730, Abcam) at 4 °C. This was followed by the treatment of lysates with PureProteome™ Protein A/G Mix Magnetic Beads (Millipore) for 2 h. Next, the agarose beads were rinsed using ice-cold PBS and the bound proteins were eluted. Western blot analysis was then performed to evaluate both the interaction between TRAF6 and Beclin-1, and the extent of of K63-linked ubiquitination of Beclin-1. The antibodies employed in this assay included anti-TRAF6 (1:2000, ab33915, Abcam) and anti-Ubiquitin (linkage-specific K63) (1:1000, ab179434, Abcam).

### Statistical analysis

All collected data underwent comprehensive statistical analysis and were graphically presented using GraphPad Prism 8.0.1 software (GraphPad Software, San Diego, CA, USA). Measurement data were expressed as mean ± standard deviation (SD). For pairwise comparisons between two groups, the t-test was executed, whereas one-way analysis of variance (ANOVA) was implemented for comparisons encompassing multiple groups. Tukey's multiple comparisons test was conducted as a post hoc analysis. The significance threshold was set as *p* < 0.05.

## Results

### Bioinformatics analysis

**Fig. 1:**
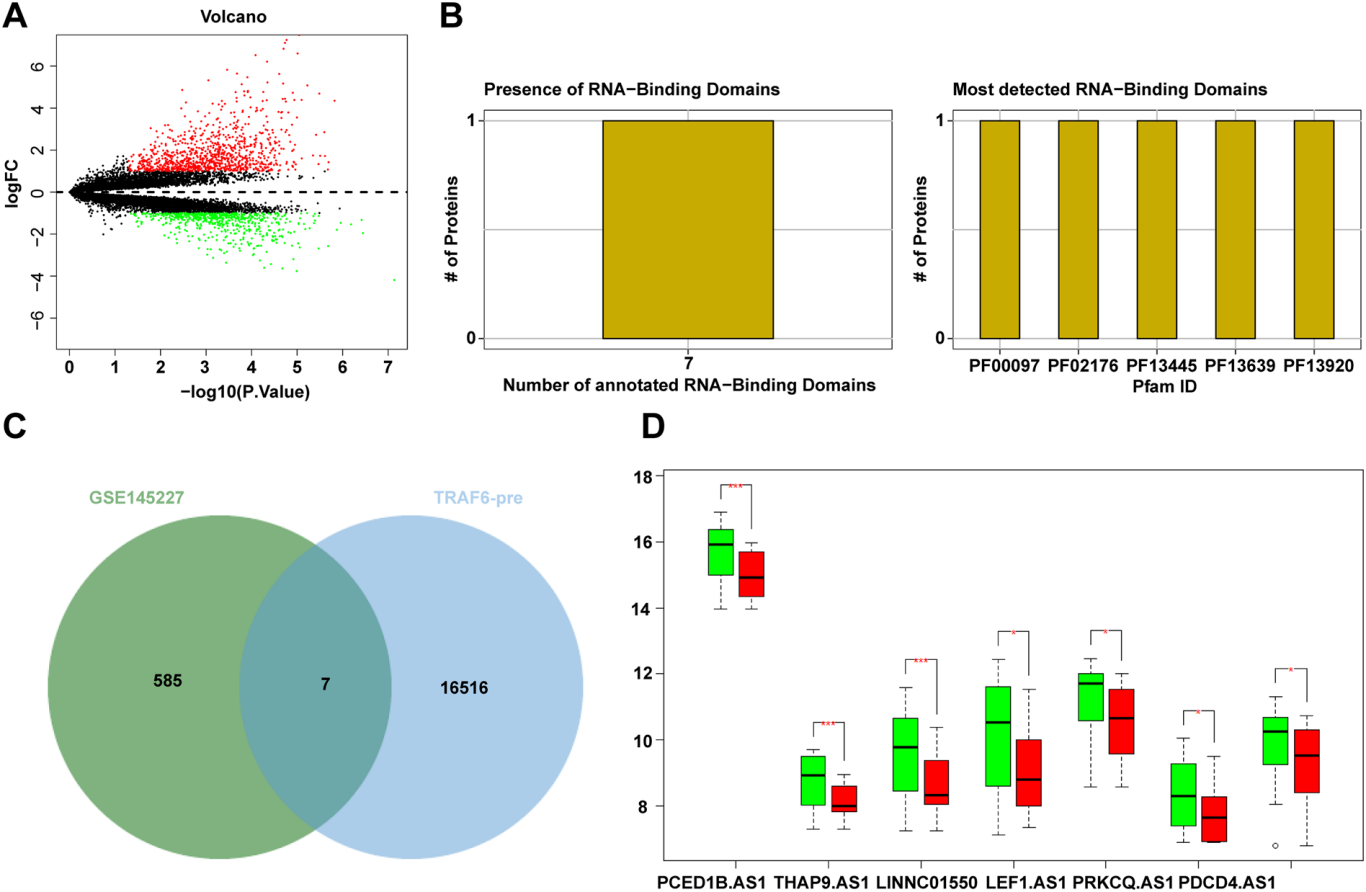
Bioinformatics analysis of abnormal expression levels of LncSICRNT1 and TRAF6 in sepsis. **A** Volcano plot of differentially-expressed genes in lncRNA microarray GSE145227 in sepsis; abscissa indicated -log10 (p.value), ordinate indicated logFC, red dots indicated significantly-upregulated genes, and green dots indicated significantly-downregulated genes; **B** Prediction of RNA-binding domain on the amino acid sequence of TRAF6; **C** Intersection of the predicted lncRNAs of TRAF6 and downregulated lncRNAs in microarray GSE145227, with the middle part indicating the intersection; **D** Differential expression of candidate lncRNAs in microarray GSE145227, with green boxplot indicating normal samples and red boxplot indicating sepsis samples. *p < 0.05, ***p < 0.005

Our investigation commenced with the differential analysis of lncRNA microarray GSE145227 extracted from sepsis through the GEO database, culminating in the identification of 1463 significantly differentially-expressed lncRNAs (Fig. 1A). Worth highlighting, TRAF6 serves as. an essential signal transducer in inflammatory signaling [31, 45, 46], displaying marked upregulation in LPS-induced SIMD [30]. Leveraging the catRAPID database, we proceeded to delineate the lncRNA-binding regions of TRAF6, unearthing multiple promising binding domains and 16,523 potential binding lncRNAs (Fig. 1B). Subsequently, through a meticulous process of intersection, where the predicted lncRNAs were matched with those that exhibited significant downregulation in sepsis, we eventually identified a focused set of 7 promising lncRNAs (Fig. 1C). Analysis of the differential expression of these 7 lncRNAs from the microarray exposed that LINC01550 (named LncSICRNT1 in this text) experienced the most substantial downregulation in sepsis (Fig. 1D, p < 0.005).

### XYT alleviated myocardial inflammation and improved cardiac function in SIMD mice by regulating LncSICRNT1 and TRAF6

**Fig. 2:**
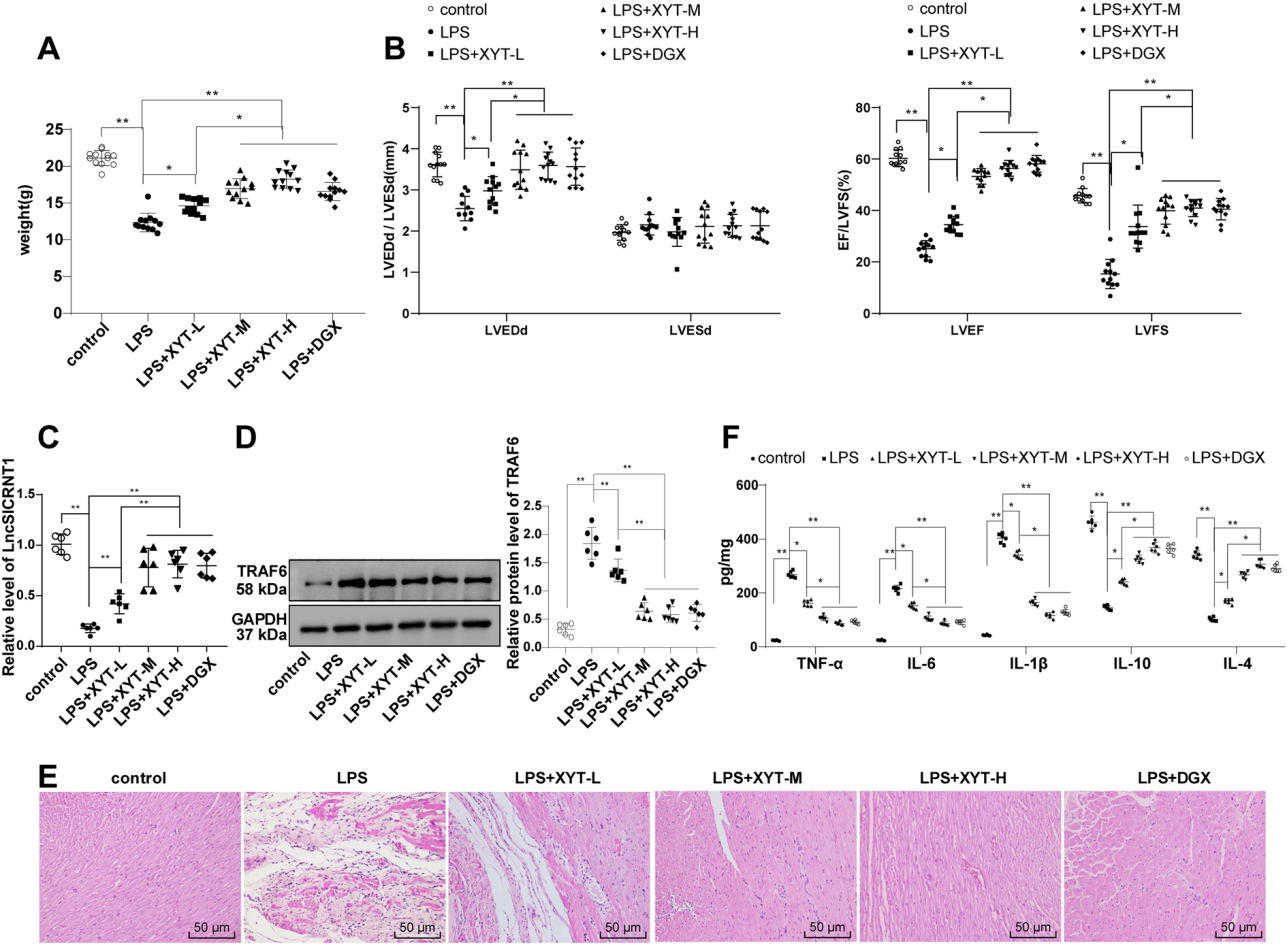
XYT alleviated myocardial inflammation and improved cardiac function in SIMD mice by regulating LncSICRNT1 and TRAF6. SIMD mice were treated with low (194 mg/kg), medium (387 mg/kg) and high (774 mg/kg) doses of XYT for 7 continuous days. **A** Mouse body weight assessment, N = 12; **B** Cardiac function evaluated by echocardiography, N = 12; **C** LncSICRNT1 expression measured by RT-qPCR, N = 6; **D** TRAF6 protein level measured by Western blot, N = 6; and the most representative protein imprints were shown in the figure; **E** Changes in mouse myocardial tissues assessed by HE staining, N = 6; and the most representative tissue staining was represented in the figure **F** Levels of pro-inflammatory cytokines TNF-α, 1L-6, IL-1β and anti-inflammatory factors IL-10 and IL-4 measured by ELISA, N = 6. Data were expressed as mean ± SD. One-way ANOVA was applied for comparisons among multiple groups, followed by the Tukey's test. *p < 0.05, **p < 0.01

After 6 h LPS (10 mg/kg) induction, C57BL/6 J mice exhibited evident symptoms like drowsiness, hair standing, disheveled, and dull, convulsions, anorexia, pyuria, and decreased movement, and a notable decline in body weight (Fig. 2A, p < 0.01). Employing echocardiography, we meticulously evaluated the cardiac function of the mice. The results revealed the significant reduction in LVEDd, LVEF and LVFS (< 50%) following the LPS induction (Fig. 2B, p < 0.01). This confluence of findings collectively validates the successful induction of SIMD in the studied mice.

To assess the impact of XYT in SIMD mice, we initiated intragastric administration of XYT at low (194 mg/kg), medium (387 mg/kg), and high (774 mg/kg) doses on the second day post-modeling. Administered twice daily for 7 consecutive days, these doses showcased remarkable improvements in sleep, hair, diet, and overall activity. Additionally, a discernible increase in body weight was observed (as depicted in Fig. 2A, with *p* < 0.05), and notably, no instances of mortality occurred within any of the treatment groups. Of significance, administration of XYT effectively ameliorated cardiac function in SIMD mice. This affirmative outcome is evidenced by the elevation in LVEDd, LVEF, and LVFS following XYT treatment (Fig. 2B, p < 0.05). Furthermore, our investigation entailed the evaluation of LncSICRNT1 and TRAF6 levels in mouse myocardial tissues through RT-qPCR and Western blot analysis. These examinations unveiled a significant reduction in LncSICRNT1 expression and an elevation in TRAF6 protein levels within myocardial tissues of SIMD mice. However, administration of XYT led to elevated LncSICRNT1 and decreased TRAF6 (Fig. 2C, D, p < 0.01). Subsequent to these analyses, we conducted HE staining to explore myocardial tissue changes in mice. The results demonstrated that LPS induction resulted in substantial inflammatory cell infiltration, disordered cardiomyocytes, and expanded space within myocardial tissues. Encouragingly, XYT treatment exhibited a partial reversal of these detrimental alterations (Fig. 2E). ELISA measurements unveiled a notable rise in pro-inflammatory cytokines TNF-α, IL-6, and IL-1β, and a decline in anti-inflammatory factors IL-10 and IL-4 in mouse myocardial tissues of mice subjected to LPS induction. Remarkably, administration of XYT led to reduced TNF-α, IL-6, and IL-1β levels, coupled with increased IL-10 and IL-4 levels (Fig. 2F, p < 0.05). Altogether, XYT attenuated myocardial inflammatory responses in SIMD mice by upregulating LncSICRNT1 and downregulating TRAF6, thereby improving cardiac function.

Furthermore, the therapeutic effect of XYT was found to be similar to that of digoxin. Notably, the medium and high doses of XYT displayed significantly more potent therapeutic effects compared to the low dose (*p* < 0.05), while no statistically significant difference was observed between medium and high doses (*p* > 0.05) (Fig. 2A-F). Consequently, for the subsequent experiments, mice were administered the medium dose of XYT.

### XYT inhibited macrophage M1 polarization and amelioratedd cardiac function in myocardial tissues of SIMD mice by up-regulating LncSICRNT1

**Fig. 3:**
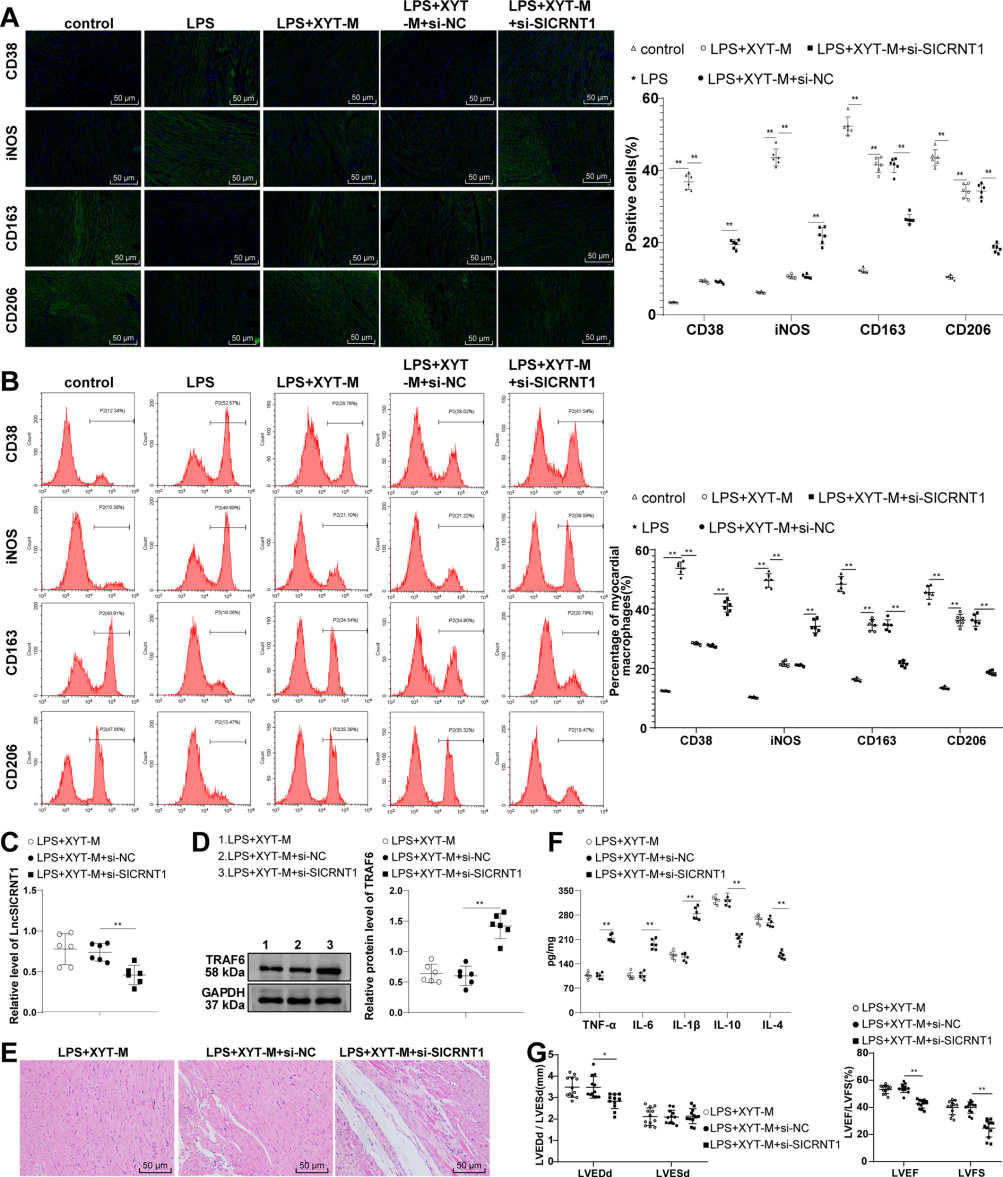
XYT suppressed macrophage M1 polarization and promoted cardiac function in myocardial tissues of SIMD mice via LncSICRNT1 upregulation. The content of cells expressing M1 macrophage surface markers CD38 + and iNOS + and M2 macrophage markers CD163 + and CD206 + in mouse myocardial tissues was detected by **A** immunofluorescence and **B** flow cytometry, N = 6; **C** LncSICRNT1 expression measured by RT-qPCR, N = 6; **D** TRAF6 protein level measured by Western blot, N = 6; and representative protein imprints were shown in the figure; **E** Changes in mouse myocardial tissuess assessed by HE staining, N = 6; and representative tissue staining was represented in the figure **F** Levels of pro-inflammatory cytokines TNF-α, 1L-6, IL-1β and anti-inflammatory factors IL-10 and IL-4 measured by ELISA, N = 6; **G** Echocardiography to evaluate cardiac function in mice, N = 12. Data were exhibited as mean ± SD. One-way ANOVA was employed for comparisons among multiple groups, followed by the Tukey's test. *p < 0.05, **p < 0.01

In the context of sepsis, the predominant inflammatory response is orchestrated by M1-polarized macrophages. Consequently, impeding M1 polarization while fostering M2 polarization plays a pivotal role in combatting sepsis sepsis [14]. Based on the previous study, we detected macrophage polarization in myocardial tissues using immunofluorescence and flow cytometry to affirm the effect of XYT on this process. The LPS group displayed an elevated population of cells expressing M1 macrophage markers CD38 + and iNOS + , coupled with a reduction in cells expressing M2 markers CD163 + and CD206 + . Following XYT treatment, the proportions of CD38 + and iNOS + cells declined, while those of CD163 + and CD206 + cells surged (Fig. 3A, B, all *p* < 0.01). Moreover, administering si-LncSICRNT1 adeno-associated virus plasmids alongside medium-dose XYT treatment led to a marked reduction in myocardial LncSICRNT1 levels (Fig. 3C, p < 0.01) and an elevation in TRAF6 protein levels (Fig. 3D, p < 0.01). These changes coincided with heightened inflammatory cell infiltration, perturbed cardiomyocytes, and widened intercellular spaces (Fig. 3E). Notably, the cellular contents of the M1 macrophage surface markers CD38 + and iNOS + were increased, while that of the M2 macrophage surface markers CD163 + and CD206 + were reduced (Fig. 3A, B, all *p* < 0.01). Additionally, levels of pro-inflammatory factors TNF-α, IL-6, and IL-1β surged, whereas anti-inflammatory factors (IL-10 and IL-4) dwindled (Fig. 3F, all *p* < 0.01). Furthermore, the reductions in LVEDd, LVEF, and LVFS were observed (Fig. 3G, all *p* < 0.05). Conjointly, these findings underscore that XYT curbed macrophage M1 polarization and promoted M2 polarization in SIMD mouse myocardial tissues, ultimately enhancing cardiac function by increasing LncSICRNT1 expression and diminishing TRAF6 protein levels.

### LncSICRNT1 was downregulated in M1 macrophages and repressed TRAF6 expression

**Fig. 4:**
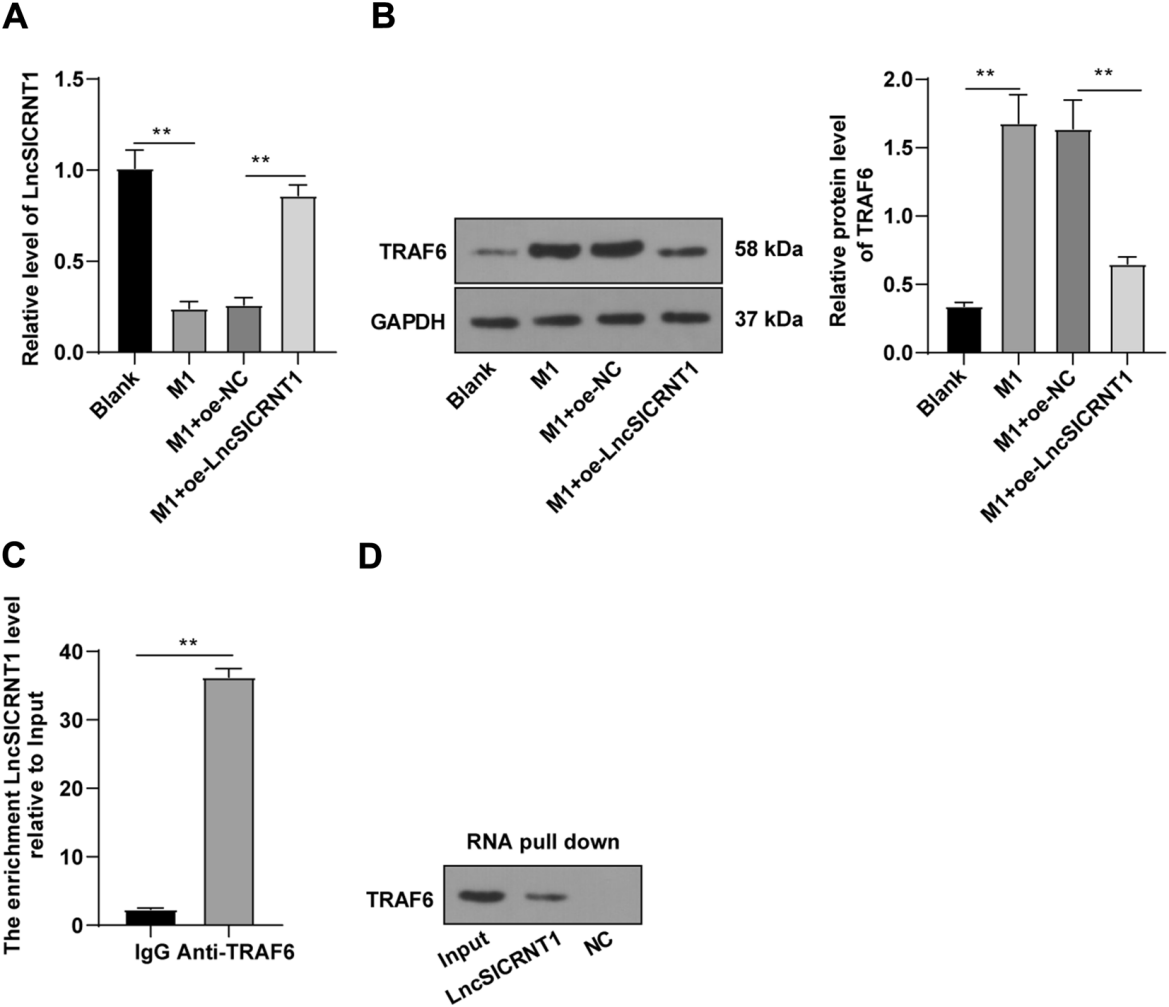
LncSICRNT1 was downregulated in M1 macrophages and repressed TRAF6 expression. The M1 macrophage model was established by stimulating BMDMs with LPS (50 ng/mL) for 24 h. **A** RT-qPCR measured LncSICRNT1 expression in cells; **B** Western blot detected TRAF6 protein level in cells; **C** RIP experiment in M1 macrophages was conducted using the anti-TRAF6 antibody or IgG (NC), and RT-qPCR detected the enrichment of LncSICRNT1 in co-precipitated RNA; **D** RNA pull-down assay in M1 macrophages was conducted using biotin-labeled LncSICRNT1 RNA and its NC, and Western blot detected the level of TRAF6. Cell experiment was repeated 3 times. Data were expressed as mean ± SD. One-way ANOVA was applied for comparisons among multiple groups, followed by the Tukey's test. **p < 0.01

To uncover the dynamic between LncSICRNT1 and TRAF6 in M1 macrophages, we initiated M1 macrophage induction by subjecting BMDMs to LPS stimulation (50 ng/mL) for 24 h. RT-qPCR assay revealed a noteworthy reduction in LncSICRNT1 expression within the M1 group compared to the blank group (Fig. 4A, p < 0.01). This trend was further underscored by Western blot results, which exhibited elevated TRAF6 protein levels in the M1 group relative to the blank group (Fig. 4B, p < 0.01). Additionally, the results of RIP and RNA pull-down assays revealed a substantial interaction between LncSICRNT1 and TRAF6 (Fig. 4C, D, p < 0.01). Upon LncSICRNT1 overexpression, the TRAF6 protein levels in the cells diminished (Fig. 4A-B, p < 0.01). In essence, LncSICRNT1 displayed diminished expression within M1 macrophages while concurrently forming a binding relationship with TRAF6, thus leading to the dampening of TRAF6 expression.

### TRAF6 induced autophagy in macrophages by mediating Beclin-1 ubiquitination

**Fig. 5:**
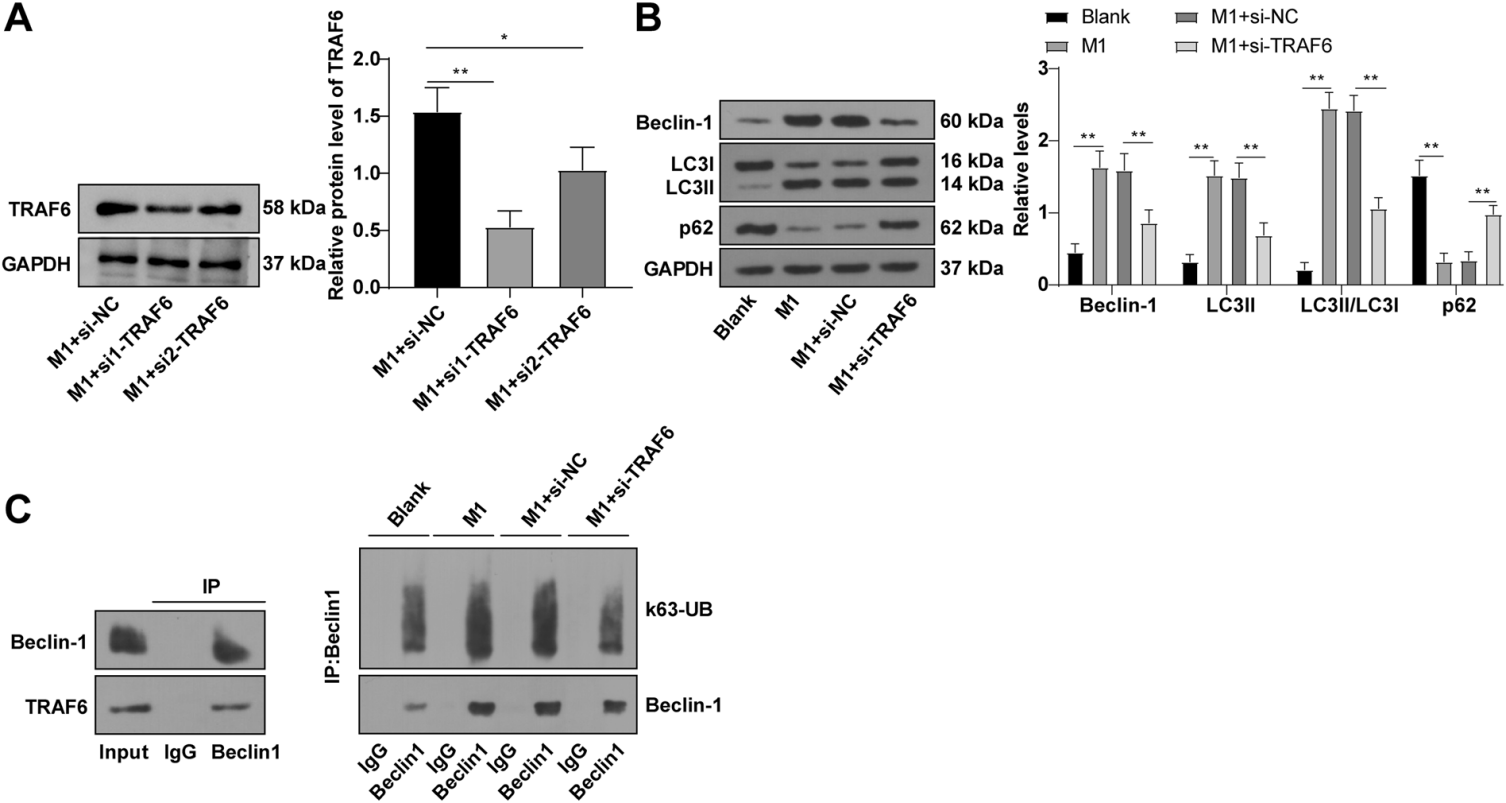
TRAF6 induced autophagy in macrophages by mediating Beclin-1 ubiquitination. (**A**, **B**) Beclin-1, LC3II, LC3II/LC3I, p62, and TRAF6 levels measured by Western blot; **C** Interaction of TRAF6 with Beclin-1 and the k63-linked polyubiquitination of Beclin-1 detected by Co-IP assay. Cell experiment was repeated 3 times. Data were exhibited as mean ± SD. One-way ANOVA was used for comparisons among multiple groups, followed by the Tukey's test. **p < 0.01

Prior research has shed light on the role of the ubiquitin ligase TRAF6 mediates K63-linked polyubiquitination of Beclin1, a process intricately tied to the initiation of autophagy [37, 47, 48]. To gain deeper insights into the impact of TRAF6 on macrophage autophagy and its subsequent influence on macrophage polarization through the modulation of Beclin1 ubiquitination in LPS-induced M1 macrophages, a targeted strategy was employed. Specifically, LPS-stimulated BMDMs were treated with si-TRAF6 to dampen TRAF6 expression. Notably, the employment of si1-TRAF6 led to a discernible reduction in intracellular TRAF6 levels (Fig. 5A, p < 0.01), thus validating its use in subsequent experimental analysis. Western blot assay unveiled distinct treads: the M1 group exhibited elevated levels of Beclin-1, LC3II, and LC3II/LC3I levels coupled with a reduced p62 protein level, as compared to the blank group. Conversely, in the M1 + si-TRAF6 group, a noteworthy pattern emerged, characterised by reduced TRAF6, Beclin-1, LC3II, and LC3II/LC3I levels, along with an elevated p62 protein level, when compared to the M1 + si-NC group (Fig. 5B, p < 0.01). Co-IP assay solidified the interaction between TRAF6 and Beclin-1 protein. Intriguingly, the K63-linked polyubiquitination of Beclin-1 exhibited a notable increase in the M1 group when contrasted with the blank group. Conversely, the M1 + si-TRAF6 group displayed a decrease in K63-linked polyubiquitination of Beclin-1 compared to the M1 + si-NC group (Fig. 5C, p < 0.01). In summary, these findings underscore the role of TRAF6 in triggering macrophage autophagy through the regulation of K63-linked polyubiquitination of Beclin-1.

### XYT promoted M2 polarization of macrophages by inhibiting autophagy

**Fig. 6:**
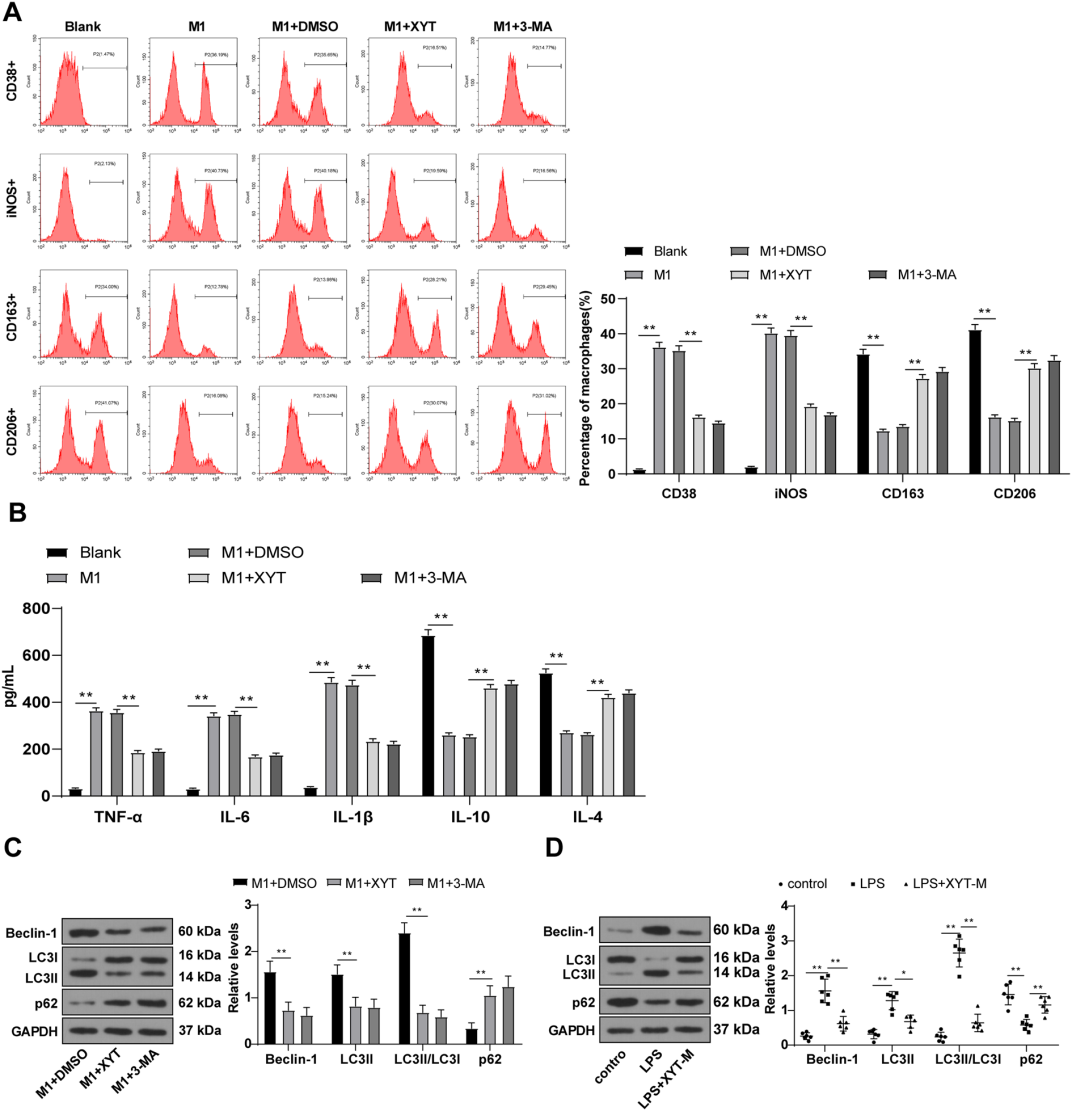
XYT promoted M2 polarization of macrophages by inhibiting autophagy. **A** Flow cytometry detected the levels of CD38 + , iNOS + , CD163 + , CD206 + cells; **B** ELISA detected the levels of TNF-α, 1L-6, IL-1β, IL-10, and IL-4 in macrophages; (**C**, **D**) Western blot detected the levels of Beclin-1, LC3II, LC3II/LC3I, and p62 in cells or mouse myocardial tissues. Cell experiment was repeated 3 times, **D**: N = 6, the most representative protein imprinting was displayed in the figure. Data were displayed as mean ± SD. One-way ANOVA was employed for comparisons among multiple groups, followed by the Tukey's test. **p < 0.01

The intricate interplay between autophagy, macrophage polarization, and apoptosis has been highlighted in prior research [49–51]. Notably, studies have indicated that hydrogen-rich saline can modulate alveolar macrophage polarization and apoptosis, offering protection against lung injury in septic rats by inhibiting autophagy [44]. In the context of LPS-induced M1 macrophages, our investigation involved treating cells with either XYT (30 μg/ml) or an equivalent amount of DMSO, while utilizing the autophagy inhibitor 3-MA (10 mM) as a positive control. A comparison between the blank group and the M1 group showed an increase in CD38 + and iNOS + cell counts, alongside a decrease in CD163 + and CD206 + cell counts. Concurrently, elevated levels of TNF-α, IL-6, and IL-1β were observed, while the levels of anti-inflammatory cytokines IL-10 and IL-4 were reduced. Remarkably, the M1 + XYT group exhibited reduced counts of CD38 + and iNOS + cells along with increased counts of CD163 + and CD206 + cells. Furthermore, the group showed a decline in Beclin-1, LC3II, and LC3II/LC3I levels, coupled with an increase in p62 protein levels. Importantly, the M1 + XYT group demonstrated alterations similar to those seen in the M1 + 3-MA group, with no significant distinctions (Fig. 6A–C, all *p* < 0.01). These results underscore the ability of XYT to foster macrophage M2 polarization by restraining macrophage autophagy. To validate this mechanism in SIMD mice, we conducted in vivo experiments using a medium dose of XYT for treatment. After LPS induction, elevated levels of Beclin-1, LC3II, and LC3II/LC3I levels, alongside a reduced p62 protein level, were detected in the mouse myocardial tissues of the mice. Strikingly, XYT treatment led to a reduction in Beclin-1, LC3II, and LC3II/LC3I levels, while enhancing the p62 protein level (Fig. 6D, all *p* < 0.01). Collectively, these findings reinforce XYT’s role in promoting macrophage M2 polarization by repressing macrophage autophagy within myocardial tissues of SIMD mice.

## Discussion

**Fig. 7:**
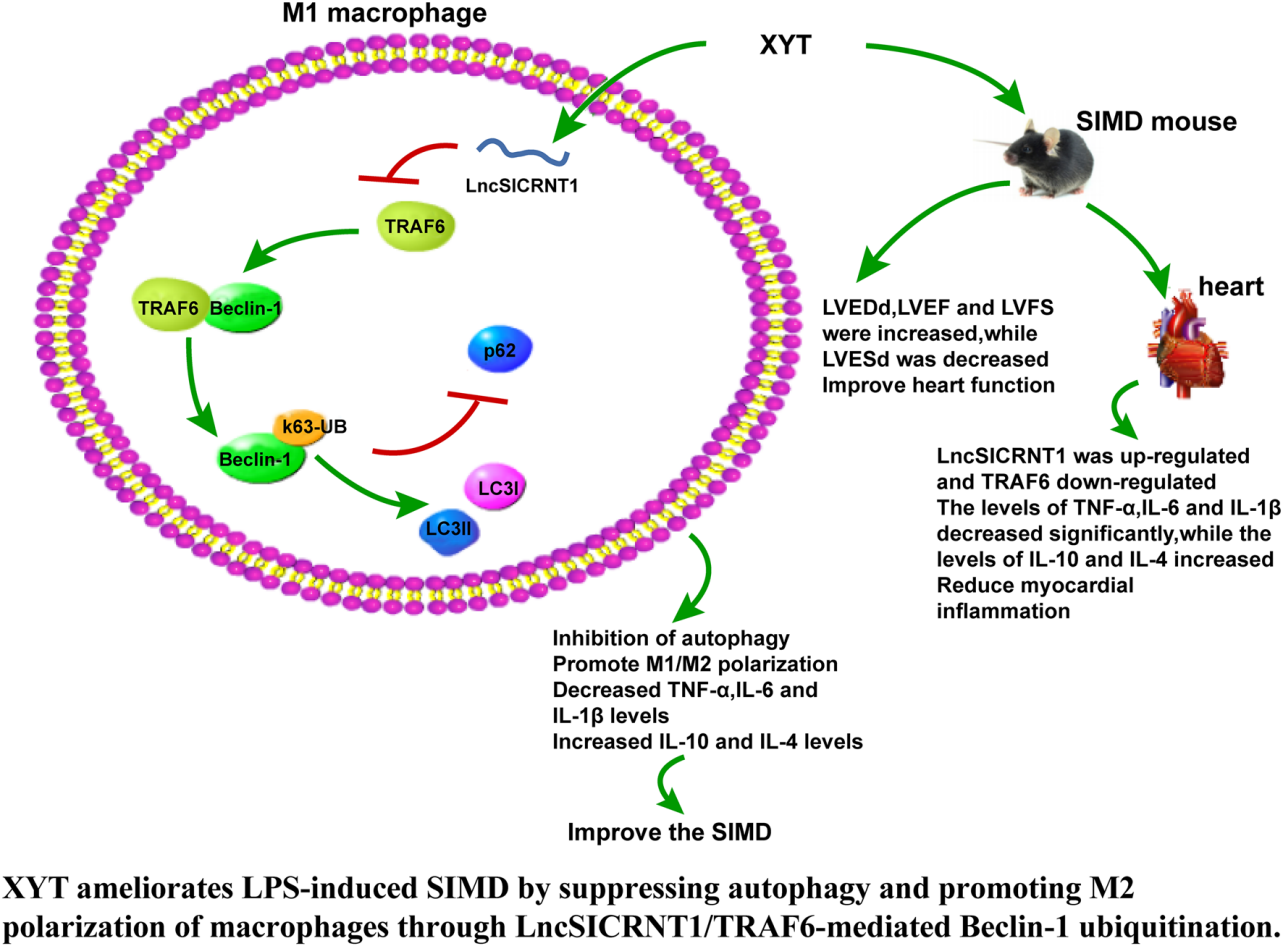
Mechanism diagram.

Sepsis is classified as an immunological response that culminates in multi-organ failure, characterised by immune system impairment due to an uncontrolled response to infection [52]. Among its severe components, SIMD stands out within the realm of sepsis-induced multi-organ failure, closely associated with adverse outcomes and heightened mortality rates [53]. Noteworthy research has unveiled the abnormal expression of 74 lncRNAs in rats experiencing sepsis-induced myocardial injury, underscoring the involvement of lncRNAs in this pathological progression [54]. Inhibiting TRAF6 has been demonstrated to mitigate sepsis-induced cardiomyopathy [55]. The realm of traditional Chinese medicine has recently emerged as a treatment avenue for heart failure, exhibiting the potential to enhance myocardial metabolism [56]. This study probed into the intricate mechanism of XYT to attenuate SIMD through interaction between LncSICRNT1 and TRAF6 (Fig. 7).

Notably, the involvement of lncRNAs and their interactions with mRNAs in the development of SIMD has been reported by Li C et al. [57]. Previous research has demonstrated that TRAF6 plays a central role as a mediator in sepsis-associated cardiomyopathy through the Akt signaling, which becomes heightened and exacerbates cardiac injury in LPS-treated mice [58]. Initially, we conducted an analysis of the microarray GSE145227 in the context of sepsis, identifying potential lncRNAs that bind to TRAF6. Subsequently, we focused on LncSICRNT1, which was significantly downregulated for further investigation. Although LncSICRNT1 has been found to be decreased in conditions like clear cell renal cell carcinoma, melanoma, and diabetic sub-dermal endothelial cells [59–61], no studies has explored its roles in SIMD. Using LPS-induced SIMD mouse models, we observed a reduction in LncSICRNT1 levels and an increase in TRAF6 expression. Upon treating SIMD mice with XYT, we observed improvements in cardiac function, accompanied by upregulated LncSICRNT1 and downregulated TRAF6. Inflammation is a well-known complement of SIMD [62], and our findings revealed that XYT improved sleep, hair, diet, and movement in mice, and also increased their body weight. Importantly, no deaths occurred in any group, and XYT alleviated myocardial tissue damage, reduced TNF-α, IL-6, and IL-1β levels, and increased IL-10 and IL-4 levels. Consistently, XYT has a traditional application in treating cardiovascular diseases, effectively curbing myocardial apoptosis, reducing inflammation, and notably enhancing cardiac function [16]. Additionally, a similar traditional Chinese medicine, Xinyin Tablet has shown to alleviate heart failure by reducing serum levels of TNF-α and IL-6 and improving cardiac ultrastructure [63]. In summary, XYT demonstrated its ability to mitigate myocardial inflammation and enhance cardiac function in SIMD mice by regulating the LncSICRNT1/TRAF6 axis.

M1 macrophages belong to the pro-inflammatory phenotype, characterized by heightened levels of pro-inflammatory cytokines including IL-1β, IL-6, and TNF-α. Importantly, excessive and sustained activation of M1 macrophages can trigger a severe inflammatory response, prompting the use of M1 polarization inhibition as a therapeutic approach for various immune diseases [64]. In contrast, M2 macrophages play a role in anti-inflammatory responses and tissue remodeling [65, 66]. Shifting macrophages towards the M1 phenotype could worsen SIMD [67], while M2 polarization can attenuate the deterioration of myocardial function [68]. Our findings notably revealed that XYT restrains M1 polarization in macrophages while promoting M2 polarization in SIMD mouse myocardial tissues. This, in turn, bolsters cardiac function by upregulating LncSICRNT1 expression and downregulating TRAF6 protein levels. Interestingly, other traditional Chinese medicine like baicalin and salvianolic acid B also foster M2 polarization and improve cardiac function [69, 70]. Significantly, our study was the first to elucidate this efficacy mechanism of XYT.

Subsequently, we subjected bone marrow-derived macrophages (BMDMs) to LPS stimulation to induce the M1 phenotype for in vitro experimentation. Our results showed a reduction in LncSICRNT1 levels in M1 macrophages, concomitant with inhibited TRAF6 expression. Given TRAF6’s essential role in macroautophagy/autophagy activation triggered by TLR4 signaling, and the established connection between autophagy and sepsis-induced cardiomyopathy [32, 71], we proceeded to treat LPS-induced BMDMs with si-TRAF6. Our subsequent findings revealed that silencing TRAF6 led to a decrease in Beclin-1, LC3II, and LC3II/LC3I levels, while increasing p62 protein levels, and diminishing K63-linked polyubiquitination of Beclin-1 in M1 macrophages. In a study by Lee NR et al., the RIG-I-MAVS-TRAF6 axis was identified as an inducer of K63-linked polyubiquitination of Beclin-1, playing a pivotal role in in triggering autophagy [47]. Moreover, excessive autophagy probably could exert detrimental effects on cellular health and cardiac function in SIMD mice [53]. Autophagy has been recognized to exert an essential role in immunity and inflammation [72, 73], influencing cytokine production, inflammasome activation, and macrophage polarization [74]. Acknowledging the pivotal role of autophagy, we treated LPS-induced BMDMs with the autophagy inhibitor 3-MA. Strikingly, our findings demonstrated that XYT yielded effects similar to 3-MA, effectively repressing autophagy and inflammation, blocking M1 macrophage polarization, and enhancing M2 polarization. Notably, trichostatin A has been shown to induce the M2 macrophage phenotype through heightened autophagy, resulting in the attenuation of systemic inflammation and improved survival in mice with polymicrobial sepsis [35]. Likewise, our findings demonstrated that XYT accelerated macrophage M2 polarization by modulating macrophage autophagy. This mechanistic insight was further validated in vivo (Additional file 2).

To conclude, this study breaks new ground by identifying the downregulation of t LncSICRNT1 (LINC01550) in myocardial tissues of SIMD mice. Importantly, it presents XYT as a mitigating agent for SIMD by enhancing LncSICRNT1 in myocardial macrophages of SIMD mice. This is achieved through the inhibition of TRAF6-mediated K63-linked ubiquitination of Beclin-1, effectively restraining macrophage autophagy, and fostering macrophage M2 polarization. It’s essential to acknowledge a significant limitation: the explored mechanism has solely been investigated at the animal and cellular levels. It’s essential to acknowledge a significant limitation: the explored mechanism has solely been investigated. Moving forward, future investigations should focus on determining lncRNA expression in the peripheral blood of SIMD patients and uncovering other potential mechanisms through which XYT can ameliorate SIMD.

### Supplementary Information

**Additional file 1: Figure S1.** The schematic diagram of in vivo experiment.

**Additional file 2: Table S1.** Raw data of LVEDd, LVESd, LVEF, and LVFS.

## Data Availability

All data generated or analysed during this study are included in this article. Further enquiries can be directed to the corresponding author.

## References

[1] Malavika M, Sanju S, Poorna MR (2022). Role of myeloid derived suppressor cells in sepsis. Int Immunopharmacol.

[2] Caraballo C, Jaimes F (2019). Organ dysfunction in sepsis: an ominous trajectory from infection to death. Yale J Biol Med.

[3] Atici AE, Arabaci Tamer S, Levent HN (2022). Neuropeptide W Attenuates oxidative multi-organ injury in rats induced with intra-abdominal sepsis. Inflammation.

[4] Suzuki T, Suzuki Y, Okuda J (2017). Sepsis-induced cardiac dysfunction and beta-adrenergic blockade therapy for sepsis. J Intensive Care.

[5] Zheng Z, Ma H, Zhang X (2017). Enhanced glycolytic metabolism contributes to cardiac dysfunction in polymicrobial sepsis. J Infect Dis.

[6] Zaky A, Deem S, Bendjelid K, Treggiari MM (2014). Characterization of cardiac dysfunction in sepsis: an ongoing challenge. Shock.

[7] Levy MM, Evans LE, Rhodes A (2018). The surviving sepsis campaign bundle: 2018 update. Intensive Care Med.

[8] Rhodes A, Evans LE, Alhazzani W (2017). Surviving sepsis campaign: international guidelines for management of sepsis and septic shock: 2016. Intensive Care Med.

[9] Ramachandran G (2014). Gram-positive and gram-negative bacterial toxins in sepsis: a brief review. Virulence.

[10] Gordon S, Martinez FO (2010). Alternative activation of macrophages: mechanism and functions. Immunity.

[11] Coppo M, Chinenov Y, Sacta MA, Rogatsky I (2016). The transcriptional coregulator GRIP1 controls macrophage polarization and metabolic homeostasis. Nat Commun.

[12] Mantovani A, Biswas SK, Galdiero MR, Sica A, Locati M (2013). Macrophage plasticity and polarization in tissue repair and remodelling. J Pathol.

[13] Zhang N, Feng H, Liao HH (2018). Myricetin attenuated LPS induced cardiac injury in vivo and in vitro. Phytother Res.

[14] Xu F, Ma Y, Huang W (2020). Typically inhibiting USP14 promotes autophagy in M1-like macrophages and alleviates CLP-induced sepsis. Cell Death Dis.

[15] Wang X, Meng H, Wang Q (2020). Baoyuan decoction ameliorates apoptosis via AT1-CARP signaling pathway in H9C2 cells and heart failure post-acute myocardial infarction rats. J Ethnopharmacol.

[16] Wang J, Deng B, Liu J (2021). Xinyang Tablet inhibits MLK3-mediated pyroptosis to attenuate inflammation and cardiac dysfunction in pressure overload. J Ethnopharmacol.

[17] Li CX, Liu Y, Zhang YZ, Li JC, Lai J (2022). Astragalus polysaccharide: a review of its immunomodulatory effect. Arch Pharm Res.

[18] Sha W, Zhao B, Wei H (2023). Astragalus polysaccharide ameliorates vascular endothelial dysfunction by stimulating macrophage M2 polarization via potentiating Nrf2/HO-1 signaling pathway. Phytomedicine.

[19] Tian L, Zhao JL, Kang JQ (2021). Astragaloside IV alleviates the experimental DSS-induced colitis by remodeling macrophage polarization through STAT signaling. Front Immunol.

[20] Teng YY, Zou ML, Liu SY (2022). Dual-action icariin-containing thermosensitive hydrogel for wound macrophage polarization and hair-follicle neogenesis. Front Bioeng Biotechnol.

[21] Lai X, Huang X, Zeng Y (2013). Protective effect of anhydroicaritin against peritonitis in mice. Xi Bao Yu Fen Zi Mian Yi Xue Za Zhi.

[22] Meng J, Zhou C, Zhang W (2019). Stachydrine prevents LPS-induced bone loss by inhibiting osteoclastogenesis via NF-kappaB and Akt signalling. J Cell Mol Med.

[23] Jiang T, Ren K, Chen Q (2017). Leonurine prevents atherosclerosis via promoting the expression of ABCA1 and ABCG1 in a Ppargamma/Lxralpha signaling pathway-dependent manner. Cell Physiol Biochem.

[24] Liu Y, Duan C, Chen H (2018). Inhibition of COX-2/mPGES-1 and 5-LOX in macrophages by leonurine ameliorates monosodium urate crystal-induced inflammation. Toxicol Appl Pharmacol.

[25] Xing PC, An P, Hu GY, Wang DL, Zhou MJ (2020). LncRNA MIAT promotes inflammation and oxidative stress in sepsis-induced cardiac injury by targeting miR-330-5p/TRAF6/NF-kappaB Axis. Biochem Genet.

[26] Dong R, Zhang B, Tan B, Lin N (2021). Long non-coding RNAs as the regulators and targets of macrophage M2 polarization. Life Sci.

[27] Zhou L, Han C, Liu Y (2022). Astragalus membranaceus and Salvia miltiorrhiza ameliorate hypertensive renal damage through lncRNA-mRNA coexpression network. Biomed Res Int.

[28] Wang G, Zhang L, Shen H, Hao Q, Fu S, Liu X (2021). Up-regulation of long non-coding RNA CYTOR induced by icariin promotes the viability and inhibits the apoptosis of chondrocytes. BMC Complement Med Ther.

[29] Liu S, Xu DS, Li M (2021). Icariin attenuates endothelial-mesenchymal transition via H19/miR-148b-3p/ELF5 in ox-LDL-stimulated HUVECs. Mol Ther Nucleic Acids.

[30] An R, Feng J, Xi C, Xu J, Sun L (2018). miR-146a attenuates sepsis-induced myocardial dysfunction by suppressing IRAK1 and TRAF6 via targeting ErbB4 expression. Oxid Med Cell Longev.

[31] Wu H, Lu XX, Wang JR (2019). TRAF6 inhibits colorectal cancer metastasis through regulating selective autophagic CTNNB1/beta-catenin degradation and is targeted for GSK3B/GSK3beta-mediated phosphorylation and degradation. Autophagy.

[32] Min Y, Kim MJ, Lee S, Chun E, Lee KY (2018). Inhibition of TRAF6 ubiquitin-ligase activity by PRDX1 leads to inhibition of NFKB activation and autophagy activation. Autophagy.

[33] Singh AK, Pandey RK, Shaha C, Madhubala R (2016). MicroRNA expression profiling of Leishmania donovani-infected host cells uncovers the regulatory role of MIR30A-3p in host autophagy. Autophagy.

[34] Boutouja F, Brinkmeier R, Mastalski T, El Magraoui F, Platta HW (2017). Regulation of the tumor-suppressor BECLIN 1 by distinct ubiquitination cascades. Int J Mol Sci..

[35] Cui SN, Chen ZY, Yang XB (2019). Trichostatin A modulates the macrophage phenotype by enhancing autophagy to reduce inflammation during polymicrobial sepsis. Int Immunopharmacol.

[36] Qiu P, Liu Y, Zhang J (2019). Review: the role and mechanisms of macrophage autophagy in sepsis. Inflammation.

[37] Shi CS, Kehrl JH (2010). TRAF6 and A20 regulate lysine 63-linked ubiquitination of Beclin-1 to control TLR4-induced autophagy. Sci Signal..

[38] Peng Q, Xu H, Xiao M, Wang L (2020). The small molecule PSSM0332 disassociates the CRL4A(DCAF8) E3 ligase complex to decrease the ubiquitination of NcoR1 and inhibit the inflammatory response in a mouse sepsis-induced myocardial dysfunction model. Int J Biol Sci.

[39] Chen X, Liu X, Dong R, Zhang D, Qin S (2021). A retrospective observational study of the association between plasma levels of interleukin 8 in 42 patients with sepsis-induced myocardial dysfunction at a single center between 2017 and 2020. Med Sci Monit.

[40] Chen W, Gao G, Yan M, Yu M, Shi K, Yang P (2021). Long noncoding RNA MAPKAPK5-AS1 promoted lipopolysaccharide-induced inflammatory damage in the myocardium by sponging microRNA-124-3p/E2F3. Mol Med.

[41] Wang L, Li Y, Wang X (2020). GDF3 protects mice against sepsis-induced cardiac dysfunction and mortality by suppression of macrophage pro-inflammatory phenotype. Cells..

[42] Chen J, Purvis GSD, Collotta D (2020). RvE1 attenuates polymicrobial sepsis-induced cardiac dysfunction and enhances bacterial clearance. Front Immunol.

[43] Ma PF, Gao CC, Yi J (2017). Cytotherapy with M1-polarized macrophages ameliorates liver fibrosis by modulating immune microenvironment in mice. J Hepatol.

[44] Qiu P, Liu Y, Chen K, Dong Y, Liu S, Zhang J (2021). Hydrogen-rich saline regulates the polarization and apoptosis of alveolar macrophages and attenuates lung injury via suppression of autophagy in septic rats. Ann Transl Med.

[45] Ishida T, Mizushima S, Azuma S (1996). Identification of TRAF6, a novel tumor necrosis factor receptor-associated factor protein that mediates signaling from an amino-terminal domain of the CD40 cytoplasmic region. J Biol Chem.

[46] Cao Z, Xiong J, Takeuchi M, Kurama T, Goeddel DV (1996). TRAF6 is a signal transducer for interleukin-1. Nature.

[47] Lee NR, Ban J, Lee NJ (2018). Activation of RIG-I-mediated antiviral signaling triggers autophagy through the MAVS-TRAF6-Beclin-1 Signaling Axis. Front Immunol.

[48] Du M, Yuan L, Tan X (2017). The LPS-inducible lncRNA Mirt2 is a negative regulator of inflammation. Nat Commun.

[49] Ip WKE, Hoshi N, Shouval DS, Snapper S, Medzhitov R (2017). Anti-inflammatory effect of IL-10 mediated by metabolic reprogramming of macrophages. Science.

[50] Hu R, Chen ZF, Yan J (2014). Complement C5a exacerbates acute lung injury induced through autophagy-mediated alveolar macrophage apoptosis. Cell Death Dis.

[51] Chang CP, Su YC, Lee PH, Lei HY (2013). Targeting NFKB by autophagy to polarize hepatoma-associated macrophage differentiation. Autophagy.

[52] Doganyigit Z, Eroglu E, Akyuz E (2022). Inflammatory mediators of cytokines and chemokines in sepsis: From bench to bedside. Hum Exp Toxicol.

[53] Sang Z, Zhang P, Wei Y, Dong S (2020). miR-214-3p attenuates sepsis-induced myocardial dysfunction in mice by inhibiting autophagy through PTEN/AKT/mTOR pathway. Biomed Res Int.

[54] Zhang TN, Goodwin JE, Liu B (2019). Characterization of long noncoding RNA and mRNA profiles in sepsis-induced myocardial depression. Mol Ther Nucleic Acids.

[55] Cao C, Zhang Y, Chai Y (2019). Attenuation of sepsis-induced cardiomyopathy by regulation of microrna-23b is mediated through targeting of MyD88-Mediated NF-kappaB Activation. Inflammation.

[56] Shao-Mei W, Li-Fang Y, Li-Hong W (2022). Traditional Chinese medicine enhances myocardial metabolism during heart failure. Biomed Pharmacother.

[57] Li C, Liu Y, Qin J (2021). Profiles of differentially expressed long noncoding RNAs and messenger RNAs in the myocardium of septic mice. Ann Transl Med.

[58] Abdullah M, Berthiaume JM, Willis MS (2018). Tumor necrosis factor receptor-associated factor 6 as a nuclear factor kappa B-modulating therapeutic target in cardiovascular diseases: at the heart of it all. Transl Res.

[59] Zhou Z, Yang Z, Cui Y (2022). Identification and validation of a ferroptosis-related long non-coding RNA (FRlncRNA) signature to predict survival outcomes and the immune microenvironment in patients with clear cell renal cell carcinoma. Front Genet.

[60] Wan J, Liu B (2021). Construction of lncRNA-related ceRNA regulatory network in diabetic subdermal endothelial cells. Bioengineered.

[61] Chen J, Li P, Chen Z (2021). Elevated LINC01550 induces the apoptosis and cell cycle arrest of melanoma. Med Oncol.

[62] Song P, Shen DF, Meng YY (2020). Geniposide protects against sepsis-induced myocardial dysfunction through AMPKalpha-dependent pathway. Free Radic Biol Med.

[63] Liu Q, Huang X, Tian M (2020). Effectiveness and safety of Xinyin tablet in treatment of chronic heart failure: a protocol of systematic review and meta-analysis of randomized controlled trials. Medicine (Baltimore).

[64] Mantovani A, Sica A (2010). Macrophages, innate immunity and cancer: balance, tolerance, and diversity. Curr Opin Immunol.

[65] Liu PS, Wang H, Li X (2017). alpha-ketoglutarate orchestrates macrophage activation through metabolic and epigenetic reprogramming. Nat Immunol.

[66] Lawrence T, Natoli G (2011). Transcriptional regulation of macrophage polarization: enabling diversity with identity. Nat Rev Immunol.

[67] Wang Z, Liu M, Ye D (2021). Il12a deletion aggravates sepsis-induced cardiac dysfunction by regulating macrophage polarization. Front Pharmacol.

[68] Tang Y, Lin X, Chen C (2021). Nucleolin improves heart function during recovery from myocardial infarction by modulating macrophage polarization. J Cardiovasc Pharmacol Ther.

[69] Zhao M, Li F, Jian Y (2020). Salvianolic acid B regulates macrophage polarization in ischemic/reperfused hearts by inhibiting mTORC1-induced glycolysis. Eur J Pharmacol.

[70] Xu M, Li X, Song L (2020). Baicalin regulates macrophages polarization and alleviates myocardial ischaemia/reperfusion injury via inhibiting JAK/STAT pathway. Pharm Biol.

[71] Wang X, Zhu Y, Zhou Q, Yan Y, Qu J, Ye H (2021). Heat shock protein 70 expression protects against sepsis-associated cardiomyopathy by inhibiting autophagy. Hum Exp Toxicol.

[72] Matsuzawa-Ishimoto Y, Hwang S, Cadwell K (2018). Autophagy and Inflammation. Annu Rev Immunol.

[73] Deretic V, Saitoh T, Akira S (2013). Autophagy in infection, inflammation and immunity. Nat Rev Immunol.

[74] Wu MY, Lu JH (2019). Autophagy and macrophage functions: inflammatory response and phagocytosis. Cells..

