# Supplementary figures and images for: Xinyang tablet ameliorates sepsis-induced myocardial dysfunction by regulating Beclin-1 to mediate macrophage autophagy and M2 polarization through LncSICRNT1 targeting E3 ubiquitin ligase TRAF6
Source: Chin Med. 2023 Nov 2;18:143. doi: 10.1186/s13020-023-00832-7 (PMC10621131; doi:10.1186/s13020-023-00832-7)

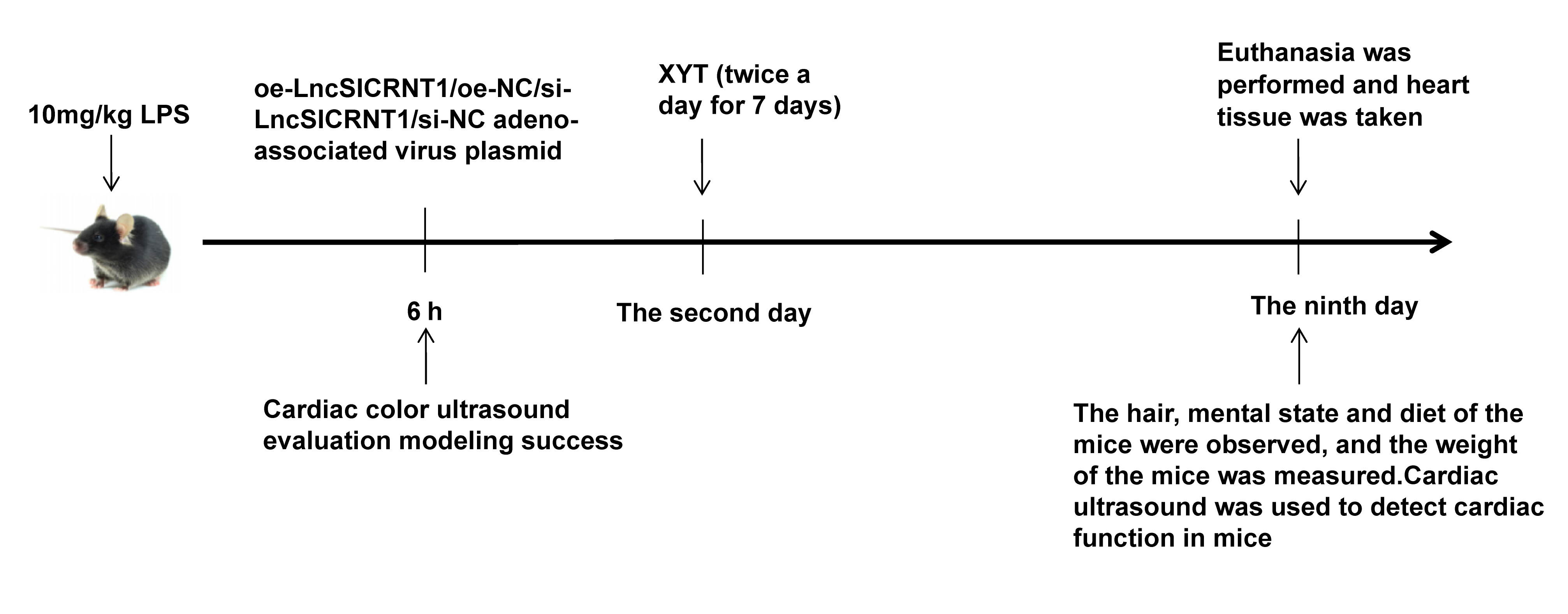

Supplement: Supplementary file 1 — Additional file 1: Figure S1. The schematic diagram of in vivo experiment. [file 13020_2023_832_MOESM1_ESM.tiff]
